# Synthesis of Silver Nanoparticles by Using *Quercus Robur* Knopper Gall Extracts

**DOI:** 10.3390/molecules30193979

**Published:** 2025-10-04

**Authors:** Vlatka Gvozdić, Zvonimir Užarević, Elvira Kovač Andrić, Vlatko Galić, Lidija Kalinić, Martina Jakovljević Kovač, Ivan Ćorić, Klara Kirchbauer, Domagoj Vidosavljević, Valentina Pavić

**Affiliations:** 1 Department of Chemistry, University of Osijek, Cara Hadrijana 8A, 31000 Osijek, Croatia; vgvozdic@kemija.unios.hr (V.G.); eakovac@kemija.unios.hr (E.K.A.); klara.kirchbauer@gmail.com (K.K.); 2 Faculty of Education, University of Osijek, Cara Hadrijana 10, 31000 Osijek, Croatia; zuzarevic@foozos.hr; 3 Department of Maize Breeding and Genetics, Agricultural Institute Osijek, Južno Predgrađe 17, 31000 Osijek, Croatia; vlatko.galic@poljinos.hr; 4 Department of Biology, University of Osijek, Cara Hadrijana 8A, 31000 Osijek, Croatia; lkalinic@biologija.unios.hr; 5 Faculty of Food Technology Osijek, University of Osijek, Franje Kuhača 18, 31000 Osijek, Croatia; mjakovljevic@ptfos.hr; 6 Department of Medicinal Chemistry, Biochemistry and Clinical Chemistry, Faculty of Medicine Osijek, University of Osijek, Josipa Huttlera 4, 31000 Osijek, Croatia; icoric@mefos.hr; 7 Department of Gynecology and Obstetrics, Faculty of Medicine Osijek, University of Osijek, Josipa Huttlera 4, 31000 Osijek, Croatia; domagoj.vidosavljevic@gmail.com

**Keywords:** silver nanoparticles, Knopper gall, *Quercus robur* L., ATR-FTIR analysis, PXRD, TEM imaging

## Abstract

Galls of the Cynipidae, such as the Knopper gall, are abnormal plant outgrowths induced by insect activity. These structures not only protect the developing larvae but also alter the biochemical properties of host plant tissues. In this study, we report the green synthesis of silver nanoparticles (AgNPs) using ethanolic extracts of *Quercus robur* Knopper galls. AgNPs were synthesized via reduction of AgNO_3_ and characterized using ATR-FTIR analysis, UV-Vis spectrophotometry, powder X-ray diffraction (PXRD), and transmission electron microscopy (TEM). The UV-Vis analysis showed a strong surface plasmon resonance (SPR) peak at 418 nm. A face-centered cubic (fcc) crystalline structure with an average crystallite size of about 12 nm was verified by PXRD patterns. TEM imaging revealed well-dispersed spherical nanoparticles, consistent with the size obtained via PXRD. ATR-FTIR analysis indicated the involvement of polyphenolic and protein-related functional groups in reduction and stabilization. The synthesized AgNPs exhibited strong growth inhibition capacity against *B. subtilis* and *S. aureus,* and moderate capacity against *E. coli* and *P. aeruginosa.* These findings highlight the potential of Knopper gall extract as a sustainable source for the eco-friendly synthesis of biologically active nanoparticles.

## 1. Introduction

Nanotechnology is transforming various fields including biomedicine, agriculture, and environmental remediation. Silver nanoparticles (AgNPs) are particularly attractive due to their size-dependent properties such as antimicrobial, antioxidant, and catalytic activities. These attributes make AgNPs valuable in biomedical applications, wastewater treatment and the development of antimicrobial coatings [1,2]. Conventional methods of AgNP synthesis typically involve toxic chemicals and energy-intensive procedures. Green synthesis, on the other hand, offers an environmentally benign alternative using natural reducing and capping agents derived from biological sources such as plant extracts, microorganisms and biomolecules [3,4]. Plant-mediated synthesis of nanoparticles offers several advantages, such as cost-effectiveness, reduced toxicity and the production of biocompatible nanoparticles with enhanced stability [5]. In AgNP synthesis, a variety of plant extracts that are high in bioactive substances such as polyphenols, flavonoids, tannins, and alkaloids have been effectively used as stabilizing and reducing agents. These phytochemicals inhibit the agglomeration of nanoparticles while promoting the reduction of silver ions (Ag^+^) to elemental silver (Ag^0^) [6]. Despite extensive research on plant-based nanoparticle synthesis, the prospect of oak knopper galls as a natural source of reducing and capping agents remains largely unexplored.

Oak knopper galls are atypical plant structures that form on the acorns of oak trees, predominantly on pedunculate oak (*Quercus robur* L.), in response to the parasitic activity of the gall wasp (*Andricus quercuscalicis* Burgsdorf) [7]. The life cycle of the gall wasp responsible for oak knopper gall formation involves two generations: a parthenogenetic generation that induces galls on pedunculate oak and a sexual generation that develops within the catkins of Turkey oak (*Quercus cerris* L.) [7,8]. Gall formation begins when females oviposit into developing acorns, triggering localized tissue changes that result in characteristic gall structures [9,10]). These galls provide both protection and nutrients for the developing larvae, which overwinter inside before emerging as adults to complete the cycle.

*Quercus robur* Knopper galls, formed in response to the parasitic activity of the gall wasp (*Andricus quercuscalicis* Burgsdorf) [7], are rich in polyphenols, tannins, and flavonoids. Despite their phytochemical richness and traditional applications in dyeing and medicine, their utility in nanoparticle synthesis has remained underexplored. These compounds exhibit strong antioxidant and metal-reducing properties, making them promising candidates for green nanotechnology applications [11]. The high tannin content of oak galls has been traditionally exploited for dyeing, medieval ink making, tanning leather, and medicinal applications. Their ability to bind metal ions and facilitate their reduction suggests their potential in nanoparticle synthesis [12]. The use of green renewable resources aims to minimize the environmental impact of chemical processes. This can be achieved through several main directions, such as choosing environmentally friendly solvents like water, using natural reducing and capping agents such as plant-based extracts and developing energy-efficient synthesis methods. These green strategies significantly reduce the impact of chemical processes on environment and their advantages are particularly pronounced in the synthesis of green nanoparticles.

Previous studies have primarily focused on the composition and biological activity of spangle galls, as well as galls from *Quercus brantii* and *Quercus infectoria*, which differ significantly in appearance from Knopper galls [13].Consequently, existing research on the antimicrobial properties of nanoparticles and gall extracts has been centered on other species of oak or different types of galls [14,15,16]. Additionally, several studies have reported the synthesis of silver nanoparticles using extracts from oak bark, leaves, and acorns; however, nanoparticle synthesis from oak gall extracts has been predominantly limited to galls of *Quercus infectoria* [17].

To the best of our knowledge, this is the first study to report the green synthesis of silver nanoparticles using ethanolic extracts of Knopper oak galls and to evaluate their antimicrobial properties. In this work, we present an environmentally friendly approach to nanoparticle synthesis inspired by the traditional use of Knopper galls in eastern Croatia, where they are locally known as “grease gall.”. These galls have long been valued in folk medicine for their perceived therapeutic effects, particularly in treating digestive ailments such as diarrhea and colic in both humans and livestock. Historically, Knopper galls were also utilized in the leather industry, especially in tanning, despite their chemical composition remaining largely uncharacterized. This body of ethnobotanical knowledge served as the foundation for our investigation into the phytochemical potential of Knopper galls as a sustainable and natural reducing and stabilizing agent for silver nanoparticle synthesis.

Knopper galls, traditionally considered a low-value or discarded by-product, are now gaining recognition as a promising resource that contributes to both the circular bioeconomy and ethnopharmacological valorization. As an underutilized natural material, their reintroduction into value chains supports the sustainable use of biological waste. Rich in tannins, phenolic compounds, and gallic acid, Knopper galls offer significant potential for bio-based applications, including natural dyes, eco-friendly pharmaceuticals, and other green products. Their valorization aligns with zero-waste principles, enhances the economic value of forestry by-products, and promotes resource efficiency within local circular development models. From an ethnopharmacological standpoint, Knopper galls have a longstanding history of traditional use for their astringent, antimicrobial, and anti-inflammatory properties. By bridging traditional knowledge with modern scientific approaches, their sustainable exploitation fosters biocultural conservation while supporting innovation, biodiversity-based development, and economic resilience.

The synthesized AgNPs were characterized using various analytical techniques, including UV-Vis spectroscopy, ATR Fourier-transform infrared (ATR-FTIR) spectroscopy, X-ray diffraction (XRD) and transmission electron microscopy (TEM), to determine their structural, morphological, and chemical properties. In addition, their antimicrobial efficacy was evaluated against selected bacterial strains to assess their potential for biomedical applications. By utilizing oak Knopper gall as a natural reducing and stabilizing agent, this study contributes to the advancement of green nanotechnology and highlights the potential of gall-derived biomolecules in environmentally friendly nanoparticle synthesis. Moreover, it demonstrates the value of an underutilized biological by-product, promoting the exploration of plant-based waste materials as sustainable resources in nanoscience. Given the increasing emphasis on environmentally friendly and sustainable approaches, the use of Knopper gall extracts for AgNP synthesis represents an innovative and sustainable strategy. Ultimately, this research connects traditional ethnobotanical knowledge with modern nanotechnology, aiming to develop a green and sustainable method for nanoparticle production.

## 2. Results and Discussion

### 2.1. AgNP Characterization Results

**Figure 1:**
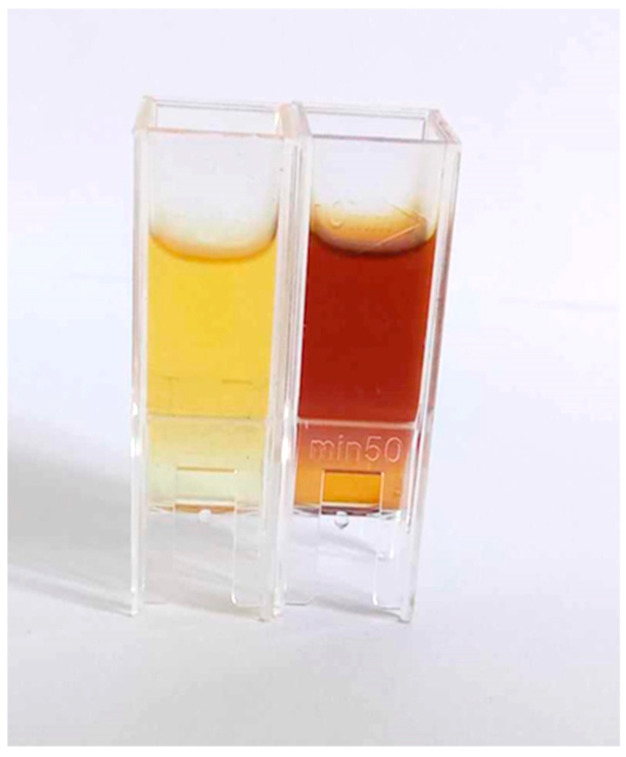
Knopper gall extract (**left**) and colloid dispersion of AgNPs (**right**).

While the color change (Figure 1) serves as a visual indicator of the progression of nanoparticle synthesis, UV-Vis spectroscopy provides a simple and reliable method for monitoring the stability of the nanoparticle solution. The formation of AgNPs during the reduction process is evidenced by a visible color transition of the reaction mixture from yellowish to dark brown, indicating the reduction of Ag⁺ ions and formation of silver nanoparticles. Figure 1 shows the color of the reaction mixture before and after the addition of 3 mM AgNO_3_ solution to the gall extract, visually confirming the successful initiation of AgNP synthesis.

**Figure 2:**
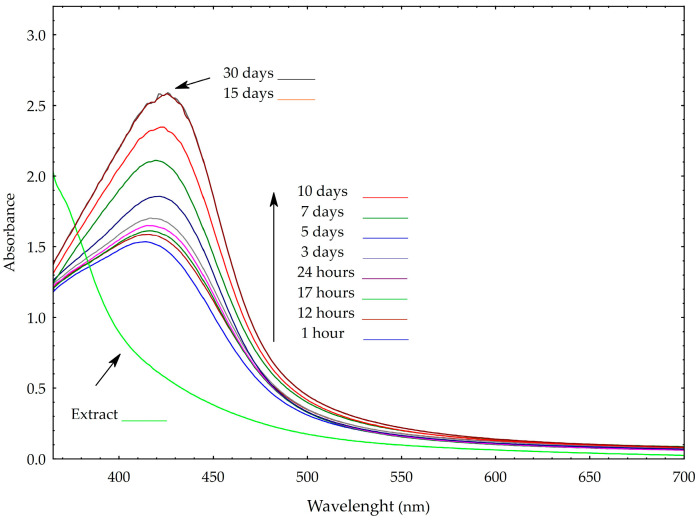
UV-Vis spectral analysis showing changes in surface plasmon resonance (SPR) intensity and peak position of silver nanoparticles over time.

Changes in the absorption band caused by Surface Plasmon Resonance (SPR) oscillations of silver electrons provide valuable insights into variations in nanoparticle size and stability [18,19,20,21]. When nanoparticles become unstable, the SPR peak typically decreases in intensity, broadens, or shifts toward longer wavelengths (bathochromic shift), often due to particle aggregation or growth in size. In some cases, the appearance of a secondary peak may indicate the formation of aggregates or non-spherical particles. As shown in Figure 2, the silver nanoparticles synthesized in this study remained stable for at least one month under ambient conditions. During this period, only a slight bathochromic shift in the SPR peak was observed- from 418 to 421 nm- suggesting minimal changes in particle size and no significant aggregation over time. Notably, spectral changes were primarily observed within the first 15 days; however, after which the spectra stabilized, indicating completion of the reduction process and successful stabilization of the nanoparticles. No additional SPR peaks were detected, implying the absence of alternative particle shapes such as hexagonal or trigonal forms. This observation was further supported by TEM analysis.

**Figure 3:**
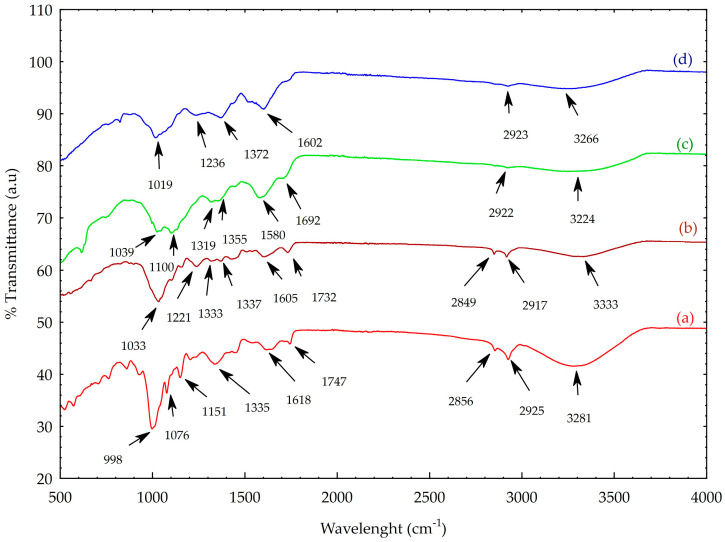
ATR-FT-IR spectra of (a) acorn kernel, (b) Knopper gall, (c) Knopper gall extract, and (d) silver nanoparticles (AgNPs).

ATR-FTIR spectroscopy was performed to identify chemical changes during the synthesis of AgNPs using the Knopper gall extract and to confirm potential interactions between the nanoparticles and bioactive compounds in the extract after synthesis. The ATR-FTIR spectra of acorn kernel, Knopper gall, gall extract, and synthesized nanoparticles are presented in Figure 3. As shown in Figure 3a,b, the galls and acorn kernel share several similar functional groups, as evidenced by the presence of comparable absorption bands. No significant differences were observed between the acorn kernel and Knopper gall spectra in the region spanning 400 cm^−1^ to approximately 1800 cm^−1^. However, in the region between 2856 (2849) and 2925 (2917), and up to 3281 (3333) cm^−1^—reflects C–H and O–H stretching vibrations– the bands are more clearly resolved in the acorn kernel spectrum.

Additionally, the spectra (Figure 3a,b) exhibit C=O stretching vibrations at 1747 (1732) cm^−1^ and 1618 (1605) cm^−1^, which are likely associated with amide groups of proteins [22,23]. A band observed at 1335 cm^−1^ in Figure 3a is linked to a cellulose-associated C–H deformation vibration [24]. These features suggest the presence of polysaccharides, polyphenols, and proteins.

The spectral region from 400 to 1151 cm^−1^ is primarily associated with carbohydrate vibrations and C-O stretching modes [25]. In the acorn kernel spectrum, bands at 1151 cm^−1^ and 1076 cm^−1^ were attributed to C-O bond stretching, indicating the presence of glucosides [26]. As shown in Figure 3b, beyond a slight band shift, the previously observed band at 1335 cm^−1^ in the acorn kernel spectrum disappears, while two new bands appear at 1333 and 1337 cm^−1^, along with an additional band at 1221 cm^−1^, possibly corresponding to C–O–C vibrations and phenolic ring breathing modes [27].

The ATR-FT-IR spectrum of AgNPs (Figure 3d) displays distinct alterations compared to that of the Knopper gall extract (Figure 3c), indicating chemical transformations during nanoparticle synthesis. For instance, the band at ~1355 cm^−1^ in the extract is absent in the AgNP spectrum, and several extract-specific bands such as those at 1692 cm^−1^ (N-H stretch of amides), 1580 cm^−1^ (C=C aromatic stretch), 1319 cm^−1^ (aromatic nitro compounds) and 1100 cm^−1^ (C=O vibration) disappear [4]. This suggests these functional groups were involved in the reduction of Ag^+^ and the capping of the resulting AgNPs [28]. The persistence of some bands in the AgNP spectrum suggests that residual biomolecules remain absorbed onto the nanoparticle surface, contributing to colloidal stability [29]. The observed band shifts, disappearances, and new peaks collectively indicate that specific biomolecular constituents in the Knopper gall extract participate in silver ion reduction and nanoparticle stabilization [14].

Most of the identified bands correspond to functional groups commonly found in polyphenols, tannins, proteins, and carbohydrates, which are known to play dual roles as reducing and capping agents in green nanoparticle synthesis [13,30].

**Figure 4:**
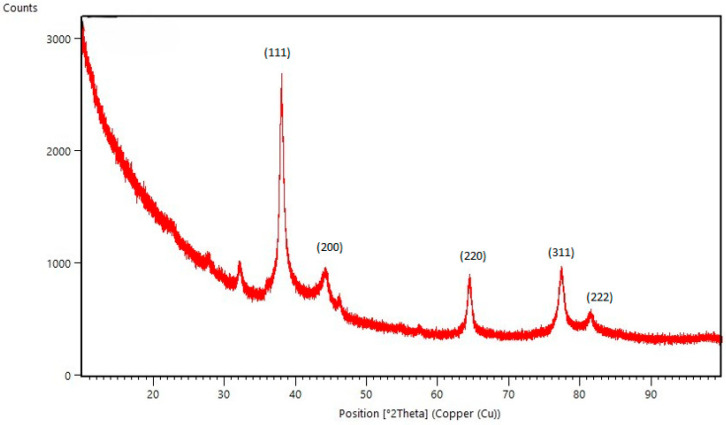
PXRD pattern of AgNPs synthesized using Knopper gall extract.

Powder X-ray diffraction (PXRD) was used to analyze the purity and confirm the crystalline nature of the synthesized AgNPs. Intense diffraction peaks corresponding to the (111), (200), (220), (311) and (222) crystal planes were observed at 38.09°, 44.10°, 64.50°, 77.42° and 81.46°, respectively, confirming the presence of face-centered cubic (fcc) crystal structure (Figure 4). The pronounced (111) reflection confirms the dominance of this plane and indicates strong crystalline ordering. The sharpness of the observed peaks reflects high crystallinity and nanoscale particle dimensions. In addition to these major reflections, a weak diffraction peak observed near 32° 2*θ* may correspond to the (111) plane of silver oxide (Ag_2_O), suggesting the presence of a minor fraction (<2%) of oxidized silver species. This likely resulted from partial surface oxidation during sample preparation or measurement under ambient conditions, as commonly reported in the literature for AgNPs synthesized via green routes [31,32,33].

The average crystallite size was calculated using Scherrer’s equation and found to be approximately 12 nm. The interplanar spacing (*d*) was 1.65 nm, while the calculated lattice parameter (*a*), was 0.4090 nm, which closely aligns with the standard lattice parameter for face-centered cubic (fcc) silver, reported as 0.4086 nm in the ICSD database (entry code 181730). This minimal deviation (Δ*a* ≈ 0.0004 nm) confirms that the synthesized nanoparticles retain the typical fcc crystal structure of metallic silver. The slight variation may be attributed to the presence of surface-bound phytochemicals from the Knopper gall extract, lattice strain, or nanoscale effects, all of which are common in green-synthesized nanoparticles. Such consistency with established crystallographic data further validates the structural integrity and successful biosynthesis of pure, crystalline AgNPs. Dislocation density (δ) and microstrain (ε) were estimated to be 7.96 × 10^11^ cm^−2^ and 3.19 × 10^−3^, respectively, indicating a low degree of crystalline defects and lattice distortion within the synthesized nanoparticles.

While surface reconstruction and lattice relaxation are known to influence the crystal structure of nanoparticles, particularly those smaller than 5 nm due to their high surface-to-volume ratio, their impact diminishes with increasing particle size. Literature reports by Huang et al. [34], suggest that these surface effects become negligible beyond this threshold. In more recent experimental work on silver nanoparticles of ~20 nm size, lattice parameters are reported close to the bulk values, with small strain and low dislocation densities, further supporting that surface-related distortions become minor at larger sizes [35].

Given that the average particle size of our synthesized AgNPs was approximately 12 nm, we consider the influence of surface reconstruction on the observed lattice parameter to be minimal. The slight variation observed (Δa ≈ 0.0004 nm) is therefore more likely attributed to lattice strain and the presence of phytochemical residues from the Knopper gall extract, rather than significant surface restructuring.

**Figure 5:**
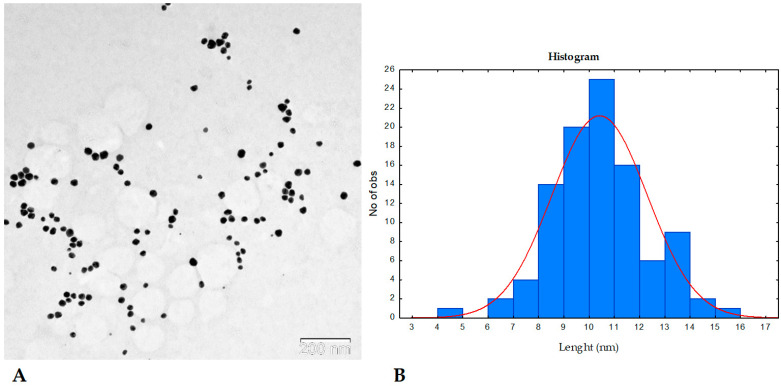
AgNPs synthesized using Knopper gall extract with a size distribution of approximately 10.76 ± 2.26 nm. (**A**) TEM image (scale bar = 200 nm). (**B**) size particle distribution histogram. The blue bars represent the frequency distribution of nanoparticle diameters, while the red line shows the Gaussian fit used to estimate the mean nanoparticle size (10.76 nm) and standard deviation (2.26 nm).

The size, shape, and distribution of the synthesized AgNPs were evaluated using transmission electron microscopy (TEM) (Figure 5A). TEM analysis revealed that the nanoparticles formed via the reduction of Ag^+^ ions with Knopper gall extract were predominantly spherical in shape, with smooth surfaces, uniform morphology, and high monodispersity. No signs of aggregation were observed. The average nanoparticle diameter was approximately 10.76 ± 2.26 nm, as determined from the size distribution histogram (Figure 5B), which aligns well with the crystallite size estimated from PXRD analysis. These findings further support the successful green synthesis of well-dispersed, nanoscale silver particles stabilized by biomolecules from the gall extract.

To date, no research has been conducted on the synthesis of nanoparticles using Knopper galls. Therefore, our findings are compared with studies involving silver nanoparticles synthesized from *Quercus infectoria* galls and other parts of oak trees, such as leaves, bark, acorns, and fruit hulls. In terms of morphology, our results are consistent with previous reports on AgNPs synthesized from oak bark, leaves, acorns, and fruit hull extracts of various *Quercus* species, all of which produced predominantly spherical nanoparticles. However, a notable distinction is the significantly smaller crystallite size obtained in our study [17,28,36,37]. For instance, silver nanoparticles synthesized from bark extracts of three different *Quercus* species exhibited spherical shapes with average diameters ranging from 45.4 to 66 nm [28]. Similarly, AgNPs biosynthesized using oak fruit hull extract were also predominantly spherical, with an average diameter of 40 nm [17]. TEM analysis of AgNPs prepared using acorn gum extract revealed highly dispersed globular nanoparticles without visible agglomeration, but with a slightly larger size (20–30 nm) than those synthesized in the present study [17]. Furthermore, silver nanoparticles synthesized via *Querqus infectoria* gall extract had an average diameter of 35 nm, with a size distribution between 27 and 68 nm—again, considerably larger than the 11 nm particles obtained in this study [14]. These comparisons highlight the effectiveness of Knopper gall extract in producing exceptionally small, monodisperse AgNPs, underscoring its potential as a valuable and underutilized source for green nanoparticle synthesis.

### 2.2. Characterization of the Knopper Gall Extracts

To evaluate the antioxidant potential of Knopper gall extracts was evaluated by three complementary assays—DPPH, FRAP, and ABTS—together with measurements of total polyphenol content (TPC) (Table 1). The extract exhibited a TPC of 72.0 mg _GAE_ g^−1^_DW_. Antioxidant activity was high, particularly in the DPPH assay (88.5 mg _Trolox_ g^−1^_DW_), followed by FRAP (48.9 mg _Trolox_ g^−1^_DW_), while the ABTS value was comparatively lower (9.4 mg _Trolox_ g^−1^_DW_).

Oak galls are known to be rich in bioactive phytochemicals, particularly hydrolysable tannins and phenolic acids, which contribute to their strong antioxidant properties, as reviewed by Banc et al. [13]. However, specific data on the antioxidant activity and TPC of Knopper galls are still limited. In our study, the measured TPC was lower than that reported values for methanolic extracts from *Andricus sternlichti* [38] and *Andricus tomentosus* galls [39]. Nevertheless, antioxidant activity values from FRAP and ABTS assays were comparable or even superior, highlighting the distinct phytochemical profile of Knopper galls. Such differences are most likely due to variations in extraction procedures, solvent systems, and the biological origin of the galls, as gall-inducing insects and host tissues (buds vs. leaves) strongly influence the chemical composition.

The HPLC analysis of the Knopper gall extract revealed a diverse profile of bioactive phenolic compounds, which likely contribute to the observed antioxidant activity. Identification of phenolic compounds was supported by comparison with authentic standards, and representative chromatograms are included in the Supplementary Materials (Figures S1–S4). As shown in Table 2, ellagic acid (13.15 ± 0.002 µg g^−1^_DW_) was the most abundant phenolic acid detected, followed by hydrolysable tannins (11.66 ± 0.03 µg _TAE_ g^−1^_DW_), which are known for their strong reducing and metal-chelating properties [40,41]. These results are in concordance with previous phytochemical studies on galls derived from other oak species, particularly *Quercus infectoria*, where ellagic acid and hydrolysable tannins were also predominant constituents [13]. Other phenolics such as epigallocatechin (2.41 ± 0.004 µg g^−1^_DW_) and its gallate derivative (1.64 ± 0.002 µg g^−1^_DW_), chrysin, herniarin, protocatechuic acid, and rosmarinic acid were detected in lower amounts. These flavonoids and coumarins have recognized antioxidant and metal-reducing capacities [42], and their combined presence suggests a synergistic contribution to the reduction of Ag⁺ ions and the stabilization of AgNPs [43].

Previous investigations have demonstrated that oak galls contain exceptionally high levels of tannins (up to 81.4%), compared to significantly lower concentrations found in oak bark (~9.1%) and leaf tissues [44]. Common constituents of galls include gallic acid, ellagic acid, and various sugars, which contribute not only to the plant’s defense system but also to broader ecological interactions [45]. The elevated tannin content, in particular, serves as a natural defense for cynipid larvae against fungal pathogens, hyperparasites, herbivores, and other environmental threats [46]. Moreover, a positive correlation has been observed between tannin concentration and gall wasp diversity or abundance, suggesting that galls serve not only as protective structures but also as chemically enriched microhabitats [47].

Despite these known attributes, phytochemical studies specifically focused on *Q. robur* Knopper galls are limited. To address this gap, our study provides new experimental evidence through HPLC analysis, confirming the presence of several phenolic compounds, and complements these findings with antioxidant activity assays. Additionally, the synthesized AgNPs displayed features comparable to those obtained from other *Quercus robur* extracts and related oak sources—namely, spherical morphology (confirmed by TEM), face-centered cubic (fcc) crystalline structure (confirmed by PXRD), and selective antibacterial activity, particularly against Gram-positive bacteria [28,48]. The rich phytochemical composition of the Knopper gall extract likely plays a crucial role in nanoparticle formation, stability, and biological performance, reinforcing its potential as a valuable resource for green nanotechnology applications.

### 2.3. Minimum Inhibitory Concentrations (MICs) of Synthesized AgNPs

The silver nanoparticles synthesized using Knopper Gall extracts exhibited notable bioactivity against the tested bacterial strains, with MIC values of 0.03125 mg mL^−1^ against *Bacillus subtilis* and 0.0625 mg mL^−1^ against *Staphylococcus aureus.* In contrast, *Escherichia coli* and *Pseudomonas aeruginosa* exhibited identical MIC values of 0.250 mg mL^−1^ (Table 3).

These findings are consistent with earlier observations that Gram-positive bacteria are generally more susceptible to AgNPs, which may be attributed to structural differences in their cell walls and greater nanoparticle uptake efficiency. The selective activity observed in our study aligns with results from other oak-derived AgNPs. For instance, AgNPs synthesized from oak fruit bark extract with particle sizes of 20–25 nm demonstrated enhanced antibacterial effects against *S. aureus* and *B. subtilis* compared to Gram-negative strains [17]. It is widely recognized that smaller AgNPs—particularly those under 50 nm, and especially in the 10–15 nm range—offer increased surface reactivity, improved membrane interaction, and greater antimicrobial potency [49,50]. With an average crystallite size of about 11 nm, the AgNPs synthesized in our work are within this ideal size range, which probably explains why they have such potent anti-Gram-positive bacterial activity.

The antimicrobial action of silver nanoparticles is believed to involve several complementary mechanisms. These include disruption of the bacterial cell membrane, leading to leakage of intracellular components [51,52]; generation of reactive oxygen species (ROS) [53], which cause oxidative damage to proteins, lipids, and nucleic acids [54,55].; and the release of Ag⁺ ions that can interact with thiol groups in enzymes and interfere with essential metabolic pathways [56,57,58]. AgNPs are also known to inhibit biofilm formation [59], which may enhance their efficacy against antibiotic-resistant strains. The observed antibacterial activity in our study is likely due to the combined effects of these mechanisms, as supported by previous literature [60,61,62,63].

While *E. coli* and *P. aeruginosa* showed the same MIC values in our study (0.250 mg mL^−1^), other research using different plant sources or synthesis conditions has reported variable outcomes. For instance, Aisida et al. [64] observed strong antibacterial activity using AgNPs synthesized from *Vernonia amygdalina* (bitterleaf) extract, with particle sizes of 2–18 nm and an MIC value of 0.080 mg mL^−1^ against *S. aureus* and coliform bacteria. Similarly, Ahmed et al. [65] reported that AgNPs synthesized using bell pepper extract and quercetin exhibited MIC values between 0.040 and 0.080 mg mL^−1^ against *E. coli*, *P. aeruginosa*, and MRSA, suggesting effective membrane disruption and oxidative stress induction as underlying antimicrobial mechanisms [65]. Variances in nanoparticle size, shape, surface chemistry, and the particular phytochemical composition of the reducing extracts can all be responsible for variances in antibacterial efficacy among studies. For example, Alsamhary et al. [66] demonstrated that smaller AgNPs (3–20 nm), demonstrated significant activity against multi-drug resistant strains like MRSA and *Klebsiella pneumoniae*, with MIC values ranging from 0.180 to 0.300 mg mL^−1^.

Overall, these results highlight the promising inhibitory efficacy of Knopper gall-mediated AgNPs, particularly against Gram-positive pathogens, and support further exploration of oak-derived biocompounds as eco-friendly agents in the development of alternative antimicrobial strategies.

### 2.4. Comparative Discussion and Future Perspectives

The current study presents a green, environmentally friendly method for synthesizing silver nanoparticles using *Quercus robur* Knopper gall extract—a phytochemically distinct material that has not been previously studied for this purpose. Compared to physical methods such as laser ablation, which produce highly pure and size-controlled nanoparticles [67], green synthesis offers simplicity, scalability, lower cost, and natural capping by biomolecules that may enhance biocompatibility and stability. In contrast, laser ablation requires expensive instrumentation [68] and does not provide intrinsic functionalization [69]. While smaller nanoparticle size is often linked to stronger antimicrobial activity due to higher surface area [66,70,71], factors such as surface chemistry, capping agents, and agglomeration also play critical roles in biological performance [72,73,74]. Our strategy prioritized preserving the native phytochemical profile of the extract under mild conditions, yielding spherical, well-dispersed AgNPs with face-centered cubic structure and potent growth inhibition activity, particularly against *Bacillus subtilis.* This suggests that the extract’s composition—not just particle size—contributes to antimicrobial efficacy.

Recent work on laser-ablated selenium and cerium nanoparticles [75,76,77] have highlighted mechanisms such as ROS generation, oxidative stress, and membrane damage in antibacterial and cytotoxic effects. Although our study focused on MIC-based evaluation of antibacterial activity, we recognize the importance of these mechanisms and plan to explore them in future work using ROS and cytotoxicity assays.

While our findings demonstrate the successful green synthesis and antibacterial potential of AgNPs using Knopper gall extract, several aspects remain beyond the scope of this study and should be considered as limitations and directions for future research. The role of individual phenolic compounds and extract fractions in the reduction and stabilization of AgNPs was not specifically investigated, and the effects of collection period, gall maturity, and geographic origin on extract composition and nanoparticle characteristics remain unexplored. Furthermore, standard operating procedures for gall collection, handling, and storage have not yet been established. Comprehensive long-term stability assessments, including zeta potential measurements, DLS-based aggregation studies, and photostability tests, were also beyond the scope of this initial investigation but are planned for future work. Additional biological assays, such as viability studies, time-kill kinetics, and biofilm inhibition tests, would also provide deeper insights into the antimicrobial mode of action of AgNPs. Addressing these limitations will be essential to fully elucidate nanoparticle formation mechanisms, ensure reproducibility, and advance Knopper gall–derived AgNPs toward practical applications.

## 3. Materials and Methods

### 3.1. Material Collection

**Figure 6:**
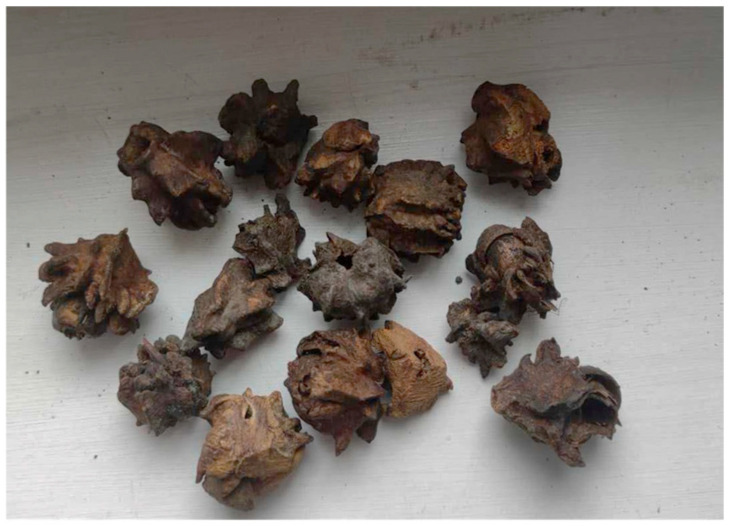
Knopper galls selected for the green synthesis of silver nanoparticles (AgNPs).

Knopper galls (Figure 6) were collected near Satnica, Eastern Croatia (45°36′54″ N; 18°29′27″ E). The samples were authenticated by Prof. Valentina Pavić and stored at the Department of Biology, University in Osijek, Croatia.

### 3.2. Extract Preparation

The galls were washed with deionized water, air-dried at room temperature (22 °C), and ground into a fine powder using a laboratory mill (MRC Laboratory Equipment Manufacturer, Holon, Israel). The powdered material was stored at the Department of Chemistry, University of Osijek, Croatia. For extract preparation, 1.3 g of gall powder was mixed with 35 mL of 70% ethanol. The container was covered and left at room temperature (24 °C) for 24 h, after which the mixture was filtered using Whatman No. 1 filter paper. The experiment was performed in triplicate.

For antioxidative activity assessment and determination of polyphenol and tannin content, approximately 0.5 g of dry gall powder was mixed with 5 mL of 70% methanol and extracted over night at −20 °C. The samples were then centrifuged using a Centric 322 A centrifuge (Tehtnica, Domel d.o.o., Železniki, Slovenia) at 10,000 RCF for 10 min at 4 °C.

### 3.3. Analyses of Antioxidative Activity and Total Polyphenols in Knopper Gall Extracts

For analyses of antioxidative activity DPPH, ABTS and FRAP assays were performed according to the protocols described in Xiao et al. in 96-wells on a microtiter plate reader (Tecan, Spark, Männedorf, Switzerland). Trolox (6-hydroxy-2,5,7,8-tetramethylchroman-2-carboxylic acid), in a range 20–100 µg/mL was used to for calibration curve. Results are expressed as equivalents of Trolox per gram of dry weight (mg _Trolox_ g^−1^_DW_).

For analyses of total soluble polyphenols (TPC) Folin- Ciocalteau assay was used with modifications described in Nemes et al. [78]. Absorbance was measured at 765 nm in 96-wells on a microtiter plate reader (Tecan, Spark, Männedorf, Switzerland). For standard curve, dilutions of gallic acid in a concentration range from 20 to 1000 µg/mL were used. Total soluble polyphenols content was expressed as milligrams of gallic acid equivalents per gram of dry weight (mg _GAE_ g^−1^_DW_).

### 3.4. HPLC Characterization of the Extracts and Hydrolysable Tannin Content

For HPLC analyses of protocatechuic acid, ellagic acid, epigallocatechin, epigallocatechin, herniarin, chrysin and rosmarinic acid, an Agilent 1260 Infinity II (Agilent, Santa Clara, CA, USA) was used as described in Kovač et al. [79]. Identification of components was carried out based on retention times and comparison of the absorption spectra in the extracts with the spectra of standards, while quantification was done based on external calibration. Standard stock solutions were prepared in methanol, and seven working concentrations (10–500 mg L^−1^) were prepared. The retention times were as follows: protocatechuic acid 10.646 min, epigallocatechin 12.613 min, epigallocatechin gallate 16.025 min, ellagic acid 31.708 min, rosmarinic acid 33.948 min, herniarin 34.035 min and chrysin 47.449 min. The linearity of the calibration curve was confirmed with R^2^ = 1.00000 for protocatechuic acid, R^2^ = 0.99505 for epigallocatechin, R^2^ = 0.99925 for epigallocatechin gallate, R^2^ = 0.95516 for ellagic acid, R^2^ = 0.99804 for rosmarinic acid, R^2^ = 0.99777 for herniarin and R^2^ = 0.99912 for chrysin. Representative HPLC chromatograms of Knopper gall extract recorded at 260, 280, and 330 nm are provided in the Supplementary Materials (Figures S1–S4). The results are expressed in µg per mg of dry weight. Authentic standards of ellagic acid (Sigma Chemical Co., St. Louis, MO, USA), protocatechuic acid, epigallocatechin, epigallocatechin gallate, and rosmarinic acid (all Sigma-Aldrich, St. Louis, MO, USA), herniarin (Sigma-Aldrich Chemie GmbH, Steinheim, Germany), and chrysin (Supelco Inc., Bellefonte, PA, USA) were used. All chemicals used were of analytical grade.

The content of hydrolysable tannins was measured on spectrophotometer (Helios γ; Thermo Spectronic, Cambridge, UK) using potassium iodate assay [80] and the protocol described in Kovač et al. [79]. The results were expressed as micrograms of tannic acid equivalent (TAE) per milligrams of dry weight (µg _TAE_ mg^−1^ _DW_).

### 3.5. AgNP Synthesis

Freshly prepared gall extract was used for the reduction of Ag^+^ to Ag^0^. Several extract-to-silver nitrate ratios (1:5, 1:10, 1:15, 1:20) were initially tested to optimize the synthesis conditions. Only at the 1:20 ratio did the UV-Vis spectra exhibit a symmetric and well-defined surface plasmon resonance (SPR) peak at 407 nm, characteristic of stable and monodisperse silver nanoparticles. At lower extract:AgNO_3_ ratios, the spectra appeared noisy and irregular, most likely due to detector saturation, as subsequent dilution yielded smooth and consistent spectra. Based on these findings, the 1:20 ratio was selected for further synthesis.

The natural pH of the obtained extract was measured to be 5.7, and no pH or temperature adjustments were made during the synthesis process, in order to preserve mild, green reaction conditions. Specifically, 30 mL of the extract was added to 600 mL of 3 mM AgNO_3_. An aliquot of the solution was taken to monitor the reduction process and to record UV-Vis spectra. The remaining solution was incubated in the dark at ambient temperature (24 °C). After 72 h, the resulting solution was centrifuged using a Centric 322 A centrifuge (Tehtnica, Domel d.o.o., Železniki, Slovenia) at 1397 RCF for 40 min at room temperature (24 °C). The resulting sediment was washed several times with deionized water, 96 h air-dried in a dark place (T = 24 °C) and used for further characterization (ATR-FIR spectroscopy, Powder X-ray Diffraction, Transmission Electron Microscopy) and the evaluation of antibacterial properties of AgNPs.

### 3.6. AgNP Characterization Procedures

#### 3.6.1. UV-Vis Spectroscopy

The reaction progress and completion were monitored using UV-Vis spectrophotometry (Shimadzu Spectrophotometer UV-1900, Shimadzu Corporation, Kyoto, Japan) in the range of 350 to 700 nm.

#### 3.6.2. ATR-FTIR Spectroscopy

To identify potential biomolecules in the extract responsible for capping and stabilizing the AgNPs, Fourier Transform-Infrared spectroscopy equipped with an Attenuated Total Reflectance (ATR) accessory was used (Spectrum Two, PerkinElmer, Waltham, MA, USA). The samples were scanned over a spectral range of 400 to 4000 cm^−1^.

For the analysis, Knopper galls were previously dried at 40 °C and then finely ground using a laboratory mill. The resulting powder was directly placed onto the ATR crystal for spectral acquisition. In the case of the acorn kernel, the outer shell was manually broken using a hammer, and a section of the inner core was cut and pressed directly onto the ATR crystal to ensure full surface contact.

#### 3.6.3. Powder X-Ray Diffraction (PXRD)

The crystalline structure of the silver nanoparticles was determined using powder X-ray diffraction (PXRD) with a Panalytical Aeris X-ray diffractometer (Malvern Panalytical Ltd., Malvern, UK).

The average crystallite size (*D*) was calculated using the Debye–Scherrer’s equation:

(1) $$D=\frac{k\lambda }{\beta cos\theta }$$

*k* is the shape factor (typically 0.9), *λ* is the X-ray wavelength (1.5406 Å), *β* is the full width at half maximum (FWHM) in radians, and *θ* is the Bragg angle.

The interplanar spacing (*d*), calculated using Bragg’s Law:

(2) 2dsinθ=nλ

The lattice parameter (*a*) was calculated using:

(3) $$a=d\surd (h^{2}+k^{2}+l^{2})$$

where *h, k* and *l* are Miller indices.

Dislocation density (δ) and microstrain (ε) were calculated using:

(4) $$\delta =\frac{1}{D^{2}},\;\;\;\;\;\;\varepsilon =\beta cos\theta /4$$

#### 3.6.4. Transmission Electron Microscopy (TEM)

The size, shape and distribution of the silver nanoparticles were examined using transmission electron microscopy (TEM) with a JEOL JEM 1200EX II instrument (Jeol Ltd., Tokyo, Japan). The particle diameters were calculated using ImageJ software (version 1.54f).

### 3.7. Determination of Minimum Inhibitory Concentrations (MICs) of Synthesized AgNPs

Following a previously described method, the minimum inhibitory concentrations (MICs) of the AgNPs synthesized using ethanolic Knopper gall extract were determined using the broth microdilution technique [81]. Growth inhibition capacity was tested against four clinically relevant human pathogens: Gram-positive (*Bacillus subtilis* and *Staphylococcus aureus*) and Gram-negative (*Escherichia coli* and *Pseudomonas aeruginosa*). The bacterial isolates, obtained from a range of clinical specimens, were provided by the Microbiology Service of the Public Health Institute of Osijek-Baranja County, Croatia. AgNPs were serially diluted (1.0 to 0.00024 mg mL^−1^) and added to bacterial suspensions in mid-logarithmic phase (5 × 10^5^ CFU mL^−1^) prepared in Mueller-Hinton broth. The assay was conducted in sterile 96-well flat-bottom polypropylene microtiter plates. For consistency, ciprofloxacin was used as a reference control and was diluted in the same manner. Additional controls included a bacterial growth control, solvent control, and a medium sterility control, all maintained under identical conditions. After a 24 h incubation at 37 °C, triphenyltetrazoliumchloride (TTC) solution was added to each well, followed by a further 3 h incubation at 37 °C. The MIC was defined as the lowest concentration of AgNPs that completely inhibited bacterial growth, indicated by the absence of a color change. All experiments were conducted in triplicate to ensure reproducibility.

## 4. Conclusions

The silver nanoparticles in this work were effectively synthesized utilizing a gentle and environmentally friendly method that used Knopper gall extract, rich in bioactive compounds and high antioxidative activity, serving as both capping and reducing agent. The synthesis method is simple, rapid and sustainable, as it generates no hazardous chemical by-products and is conducted under ambient conditions. UV-Vis spectroscopy and TEM confirmed the formation of spherical nanoparticles with an average diameter of approximately 10.76 ± 2.26 nm, consistent with the crystallite size (~12 nm) calculated from PXRD analysis. PXRD patterns further confirmed the face-centered cubic (fcc) crystalline structure of the AgNPs, with a calculated lattice parameter of 0.4090 nm, closely matching the standard value for metallic silver (0.4086 nm). This minimal deviation confirms the retention of the characteristic fcc structure and suggests that green synthesis using plant extracts can yield structurally consistent crystalline nanoparticles. The synthesized AgNPs exhibited excellent physicochemical stability and selective growth inhibition capacity, with remarkably low MIC values of 0.03125 mg mL^−1^ for *Bacillus subtilis* and 0.0625 mg mL^−1^ for *Staphylococcus aureus*. Moderate activity was observed against *Escherichia coli* and *Pseudomonas aeruginosa*, both with MIC values of 0.250 mg mL^−1^. These findings highlight the potential of Knopper gall-derived AgNPs as promising candidates for biomedical and environmental applications and support further exploration of underutilized plant-based materials in green nanotechnology. While this study focused on demonstrating feasibility, future research should address long-term stability, the role of individual phytochemicals in nanoparticle formation, and expanded biological and toxicological testing to advance these materials toward practical applications.

## Figures and Tables

**Table 1:** Total soluble polyphenol content (TPC) and antioxidative activity measured by FRAP, DPPH and ABTS assays in Knopper gall extracts. Results are presented as mean ± standard deviation (SD).

Parameter	Value
TPC mg _GAE_ g^−1^_DW_	72.0 ± 1.9
FRAP mg _Trolox_ g^−1^_DW_	48.9 ± 5.2
DPPH mg _Trolox_ g^−1^_DW_	88.5 ± 1.3
ABTS mg _Trolox_ g^−1^_DW_	9.4 ± 0.1

**Table 2:** Identified phenolic compounds in Knopper gall extract. Results are presented as mean ± standard deviation (SD) and expressed in µg g^−1^ dry weight.

Phenolic Component	Concentration (µg g^−1^_DW_)
Protochatechuic acid	0.03 ± 0.001
Ellagic acid	13.15 ± 0.002
Epigallocatechin	2.41 ± 0.004
Epigallocatechin gallate	1.64 ± 0.002
Herniarin	0.01 ± 0.000
Chrysin	0.01 ± 0.000
Rosmarinic acid	0.01 ± 0.000
Hydrolysable tannins	11.66 ± 0.030

**Table 3:** Minimum inhibitory concentrations (MICs) of silver nanoparticles synthesized using Knopper gall extracts against *Escherichia coli*, *Pseudomonas aeruginosa*, *Bacillus subtilis*, and *Staphylococcus aureus* (mg mL^−1^).

	MIC (mg mL^−1^)			
	*G+*		*G*−	
	*B. subtilis*	*S. aureus*	*E. coli*	*P. aeruginosa*
AgNPs	0.03125	0.0625	0.250	0.250
C *	0.244	1.953	1.953	0.488

* C-ciprofloxacin in µg mL^−1^.

## Data Availability

The data presented in this study are available on request from the corresponding author.

## Supplementary Materials

The following supporting information can be downloaded at: https://www.mdpi.com/article/10.3390/molecules30193979/s1. Figure S1: HPLC chromatogram of Knopper gall extract at 260 nm; Figure S2: HPLC chromatogram of Knopper gall extract at 280 nm; Figure S3: HPLC chromatogram of Knopper gall extract at 330 nm; Figure S4: Overlay of representative chromatograms.
