# Supplementary figures and images for: Synthesis of Silver Nanoparticles by Using Quercus Robur Knopper Gall Extracts
Source: Molecules. 2025 Oct 4;30(19):3979. doi: 10.3390/molecules30193979 (PMC12526430; doi:10.3390/molecules30193979)

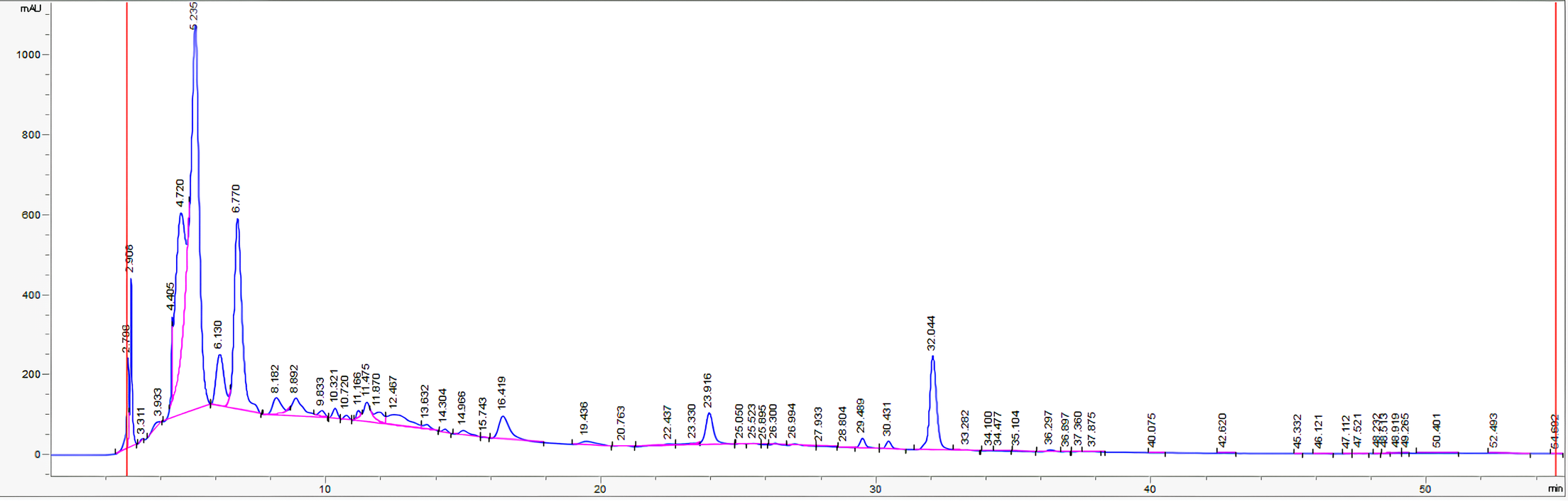

Supplement: Supplementary file 1 [file molecules-30-03979-s001.zip › Figure S2.png]

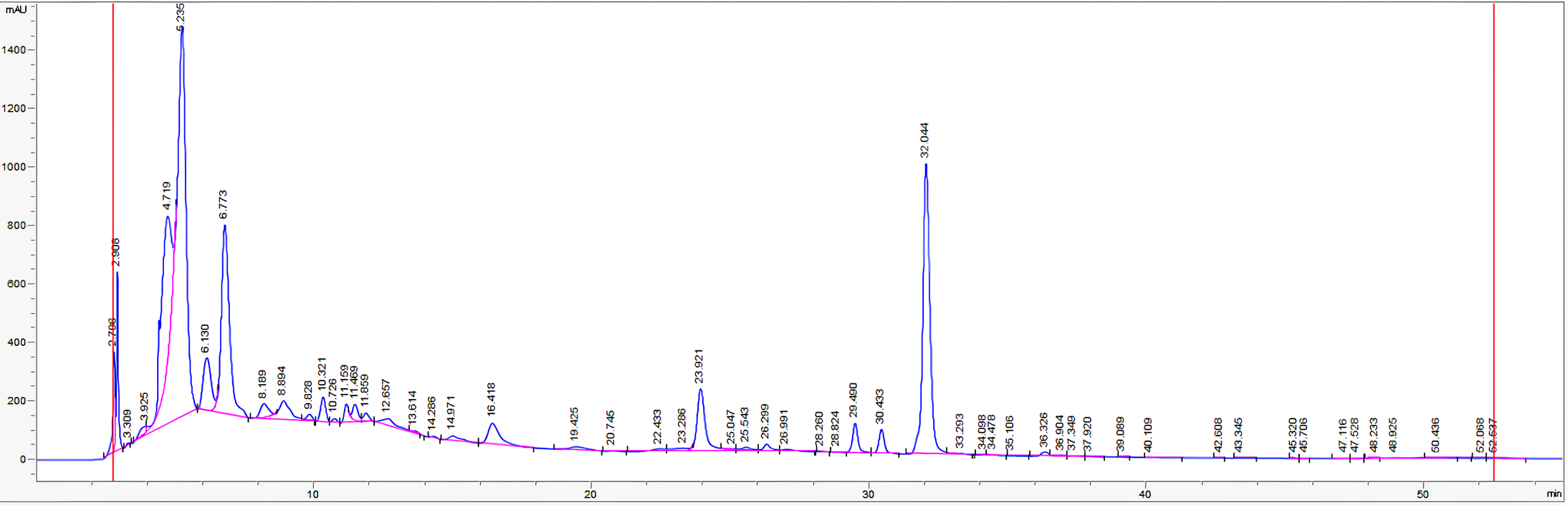

Supplement: Supplementary file 1 [file molecules-30-03979-s001.zip › Figure S1.png]

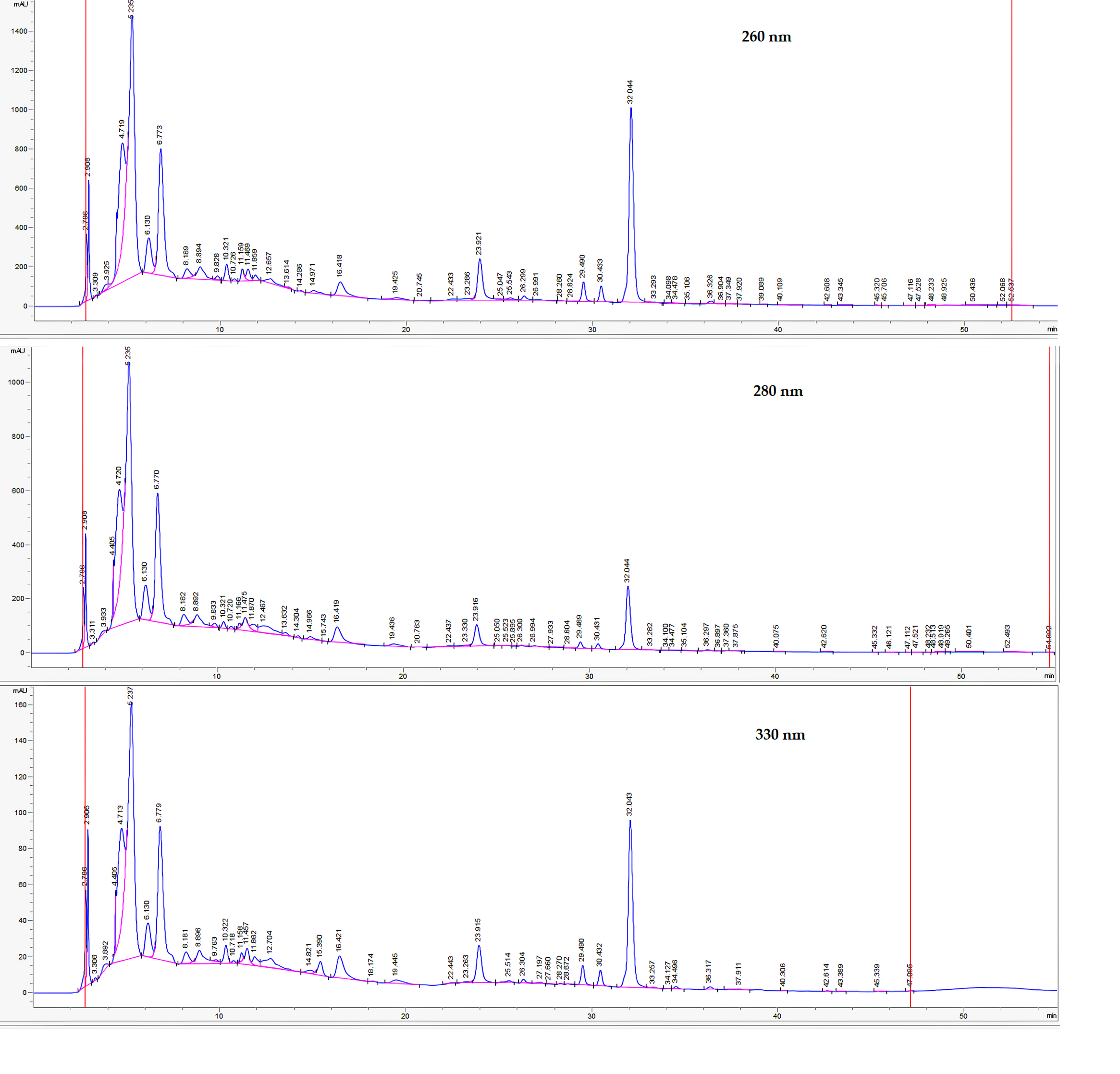

Supplement: Supplementary file 1 [file molecules-30-03979-s001.zip › Figure S4.png]

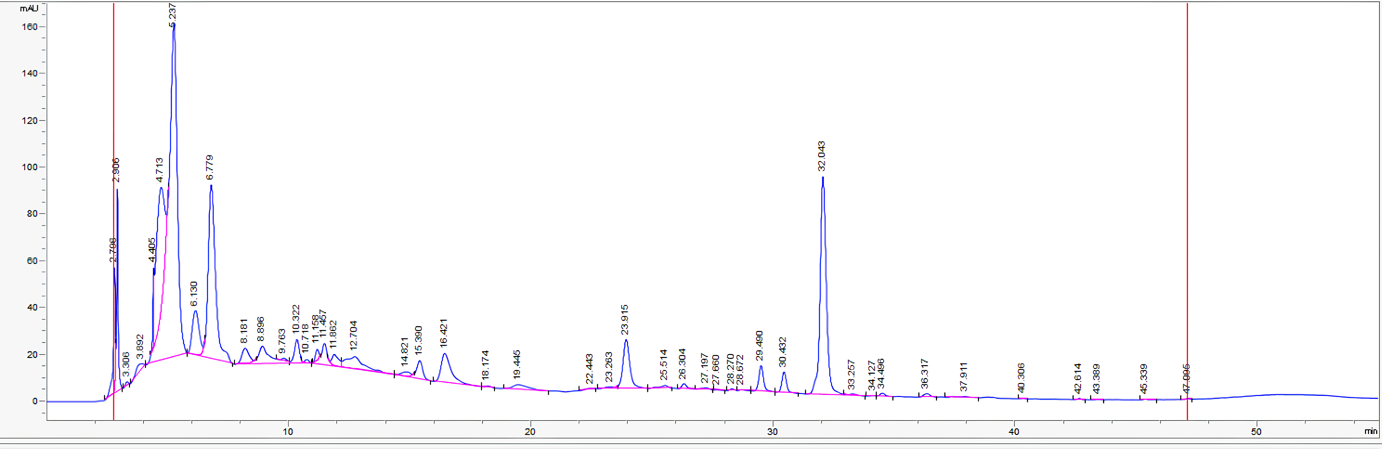

Supplement: Supplementary file 1 [file molecules-30-03979-s001.zip › Figure S3.png]
